# Towards an Inertial Sensor-Based Wearable Feedback System for Patients after Total Hip Arthroplasty: Validity and Applicability for Gait Classification with Gait Kinematics-Based Features

**DOI:** 10.3390/s19225006

**Published:** 2019-11-16

**Authors:** Wolfgang Teufl, Bertram Taetz, Markus Miezal, Michael Lorenz, Juliane Pietschmann, Thomas Jöllenbeck, Michael Fröhlich, Gabriele Bleser

**Affiliations:** 1 Junior Research Group wearHEALTH, Technische Universität Kaiserslautern, Gottlieb-Daimler-Str. 48, 67663 Kaiserslautern, Germany; taetz@cs.uni-kl.de (B.T.); miezal@cs.uni-kl.de (M.M.); lorenz@cs.uni-kl.de (M.L.); bleser@cs.uni-kl.de (G.B.); 2 Department of Sports Science, Technische Universität Kaiserslautern, Erwin-Schrödinger-Str. 57, 67663 Kaiserslautern, Germany; michael.froehlich@sowi.uni-kl.de; 3 Institut für Biomechanik, Klinik Lindenplatz, Weslarner Str. 29, 59505 Bad Sassendorf, Germany; Juliane.Pietschmann@klinik-lindenplatz.de (J.P.); Thomas.Joellenbeck@klinik-lindenplatz.de (T.J.)

**Keywords:** 3D gait analysis, inertial measurement unit, joint kinematics, machine learning, osteoarthritis, range of motion, rehabilitation, spatio-temporal parameters, support vector machine

## Abstract

Patients after total hip arthroplasty (THA) suffer from lingering musculoskeletal restrictions. Three-dimensional (3D) gait analysis in combination with machine-learning approaches is used to detect these impairments. In this work, features from the 3D gait kinematics, spatio temporal parameters (Set 1) and joint angles (Set 2), of an inertial sensor (IMU) system are proposed as an input for a support vector machine (SVM) model, to differentiate impaired and non-impaired gait. The features were divided into two subsets. The IMU-based features were validated against an optical motion capture (OMC) system by means of 20 patients after THA and a healthy control group of 24 subjects. Then the SVM model was trained on both subsets. The validation of the IMU system-based kinematic features revealed root mean squared errors in the joint kinematics from 0.24° to 1.25°. The validity of the spatio-temporal gait parameters (STP) revealed a similarly high accuracy. The SVM models based on IMU data showed an accuracy of 87.2% (Set 1) and 97.0% (Set 2). The current work presents valid IMU-based features, employed in an SVM model for the classification of the gait of patients after THA and a healthy control. The study reveals that the features of Set 2 are more significant concerning the classification problem. The present IMU system proves its potential to provide accurate features for the incorporation in a mobile gait-feedback system for patients after THA.

## 1. Introduction

Hip osteoarthritis describes a degenerative process of the cartilage at the hip joint. Pain and immobility are the common consequences leading to changed gait patterns in the affected subjects. Total hip arthroplasty (THA) is considered the most promising option once conservative therapies are exhausted [1]. However, gait abnormalities, such as asymmetries in the kinematics between implanted and non-implanted hip joints, persist even after successful THA and the consecutive rehabilitation process [2]. The literature reveals persisting changes of the joint angle kinematics of the implanted limb: rather, a higher posterior tilt of the pelvis during stance phase, an increased pelvic drop towards the non-implanted side while loading the implanted side, an increased hip internal rotation and hip adduction as well as a decreased hip extension peak and a reduced hip range of motion (ROM) in the sagittal plane [2,3,4,5,6]. According to Queen et al. [6], these asymmetries persist up to one year after the THA and they recommend a continuative physical therapy to eliminate these deviations.

Spatio-temporal gait parameters (STP) like stride length, gait velocity, etc. are further considered valuable parameters for the assessment of the outcome of THA [7,8,9]. Several studies reported a continuative reduction of stride length, step length and gait speed compared to able-bodied controls [10,11,12].

Persisting gait deviations can increase the risk of falls and interfere with the quality of life [3]. Gargiulo et al. [13] identified generalized rehabilitation methods instead of a patient specific rehabilitation process as one of the main problems.

Three-dimensional (3D) movement analysis has proven to be a useful tool for assessing the individual rehabilitation process and for comparing pre and post-operative gait in patients after THA [1,2,13,14]. Marker-based optical motion capture (OMC) systems are commonly used for gait analysis [15]. However, OMC systems cover only a limited capture volume and are not applicable in the subject’s natural environment. In the last few decades, inertial measurement unit (IMU) systems have been intensively developed [16] to address these restrictions of the common measurement systems. The 3D joint angles as well as the STP of IMU systems were evaluated in different studies [17,18,19,20,21,22,23,24,25,26,27,28].

To be of use in a clinical background, Laroche et al. [1] pointed out that 3D gait analysis must prove its ability to discriminate impaired and non-impaired gait. For that reason, various studies used different machine-learning (ML) approaches to classify the gait of different populations [1,29,30,31,32,33].

One common method concerning a classification problem is the support vector machine (SVM). The basic theory of the SVM in a classification problem including two data sets is to find the hyperplane that best separates the two groups. If a linear separation cannot be found on the input data, the data can be transformed into a higher dimensional (feature) space, using different kinds of kernel functions, like a linear, polynomial or radial basis function (RBF) kernel. Subsequently, the separation can be searched in feature space [34].

A SVM has been used for the differentiation of impaired and non-impaired gait [1,29,30,31,35]. Figueiredo et al. [29] reported that the SVM is a reliable classifier of human gait based on high-dimensional data, especially for offline walking recognition.

Lau et al. [35], who used an SVM to classify the gait of patients after a stroke with drop foot employing two IMUs on shank and foot, suggested a Gaussian RBF kernel for individual gait classification problems.

Begg et al. [30] examined the gait of young and old subjects and employed an SVM with different kernel methods trained on 2D video analysis based features to differentiate their gait patterns. The SVM classifier incorporating a linear or polynomial kernel and different numbers of features reached an area under the curve (AUC) of 0.63 to 0.83. The SVM classifier employing an RBF kernel and different numbers of features reached an AUC of 0.75 to 0.95.

Laroche et al. [1] used a SVM with linear kernel to differentiate between the gait of patients with hip osteoarthritis and healthy controls based on 12 OMC-based joint angle trajectories. They reported an accuracy of their model of approximately 88%.

However, there is a lack of studies that try to differentiate between the IMU-based 3D joint kinematics of healthy subjects and those of patients after THA. Furthermore, for that purpose, no commonly acknowledged and biomechanically interpretable features, which can also be used to provide feedback, have been defined yet.

Hence, it was the aim of this study to employ a ML algorithm to classify the gait of healthy subjects and subjects after THA based on features selected from IMU-based kinematics. Therefore, a previously reported method was used [36] to calculate purely IMU-driven gait kinematics, STP as well as joint angles.

The IMU system in question is based on gyroscope and accelerometer data and incorporates an algorithm based on an iterated extended Kalman filter approach [37,38]. That system was validated against an OMC system in previous studies among a population of young and healthy subjects [36,39,40]. The 3D joint kinematics of the lower body was evaluated during the gait and physiotherapy specific movements in [39,40]. However, only the pure technical performance of the algorithm was evaluated, relying on an OMC-based calibration, initialization, and biomechanical model.

The event detection of initial contact and terminal contact [36] and, based on that, the estimation of the STP, were evaluated using the same algorithm as in [39,40], however, adding an IMU-based calibration, an IMU-based initialization as well as a biomechanical model based on scaling according to anthropometric tables, thereby introducing an autarkic system.

Most of the ML-related articles chose their features either automatically from raw input data or from employing statistical features or other input transformations, combined with a dimensionality reduction and/or a feature selection approach; this often renders interpretability of the employed features a hard task. In the present article, however, it was the aim to introduce meaningful features that are intuitive to physicians and patients alike. Therefore, in this study the features were chosen based on the literature and expert knowledge and then calculated from among the IMU-based kinematics.

Additionally, the features in question are validated against an OMC system, thus supporting the potential usefulness of the present system in a clinical context. Furthermore, the same ML algorithm trained on IMU data is in turn trained on OMC data, to indicate the independency of the selected features from the measurement system.

## 2. Materials and Methods

### 2.1. Feature Selection

In the present study the calculated features were chosen based on the literature [2,3,4,5,6,8,9,10,11,41] and the experience of movement scientists and physical therapists within the research group.

Pietschmann et al. [41] showed that the sagittal hip joint angle, measured by means of IMUs, is an essential measure in patients after THA for providing acoustic feedback during treadmill-based gait training. The sagittal hip joint angle was further described as an important measure in patients with THA by several other authors [2,4,5,6,10,11]. Gait speed [4,8,10], maximum hip extension [2,5,6,10], maximum hip flexion [3], pelvis transversal as well as sagittal [2] and frontal [42,43] ROM, stride length [10], stride time and cadence [9] were identified as further measures of interest regarding patients after THA.

The parameters mentioned above refer to a comparison of the operated side to a healthy control as well as to the contralateral limb, i.e., as symmetry value. This was one reason for choosing the difference between operated and non-operated limb, regarding the joint kinematic-based features, if possible. However, another reason for doing so was to reduce the transfer of errors from the measurement system, e.g., calibration offsets, into the selected features. Symmetry values and the ROM are considered independent of static offsets [40].

The features defined above were then separated into two sets. First, features which are easy to measure with common gait analysis tools, e.g., two IMUs or insoles and are, therefore, widely used in the assessment of gait, i.e., STP (Set 1). Second, features which are more complex to calculate and rely on the estimation of relative segment orientations, i.e., joint kinematics (Set 2).

The defined features are summarized in Table 1.

The features in Set 1 and Set 2, respectively, were investigated regarding their correlation within the corresponding set. Therefore, the Pearson correlation coefficient (r) was calculated.

Both feature sets combined were further investigated regarding the feature importance, independently of the actual SVM classification approach. The feature importance was evaluated using a minimum redundancy maximum relevance (MRMR) algorithm [44].

### 2.2. Subjects

Twenty subjects after THA (13 females, 7 males; age 56.9 ± 8.2 years; 82.9 ± 18.9 kg and 1.74 ± 0.1 m in height) participated in the study (THA Group). All patients approximately 2 weeks after a standard cemented THA who could steadily walk at least for four minutes without support were included in the examination. All patients included in the study were allowed full weight bearing. The subjects were recruited from among the patients of the Klinik Lindenplatz (Bad Sassendorf, NRW, Germany). The study was approved by the ethical committee of the Universität Paderborn and meets the criteria of the declaration of Helsinki. After receiving all relevant study information, the participants signed an informed consent to the study including a permission to publish the data.

Additionally to the sample of 20 subjects after THA the data of 24 healthy subjects from [36] was also included (Control Group).

### 2.3. Data Acquisition

All measurements were recorded at the biomechanics laboratory of the institute of biomechanics of the Klinik Lindenplatz. Prior to the measurement and the subject preparation a gyroscope and accelerometer bias estimation was performed. Therefore, the IMUs were fixed in a rectangular box. The box was positioned once on each side for a few seconds. The IMU measurements during this procedure were recorded. The accelerometer bias estimation was performed using a spherical fitting, similar to a method used for magnetometers [45]. Additionally, the gyroscope biases were calculated as the sample means of the measured gyroscope values.

**Figure 1:**
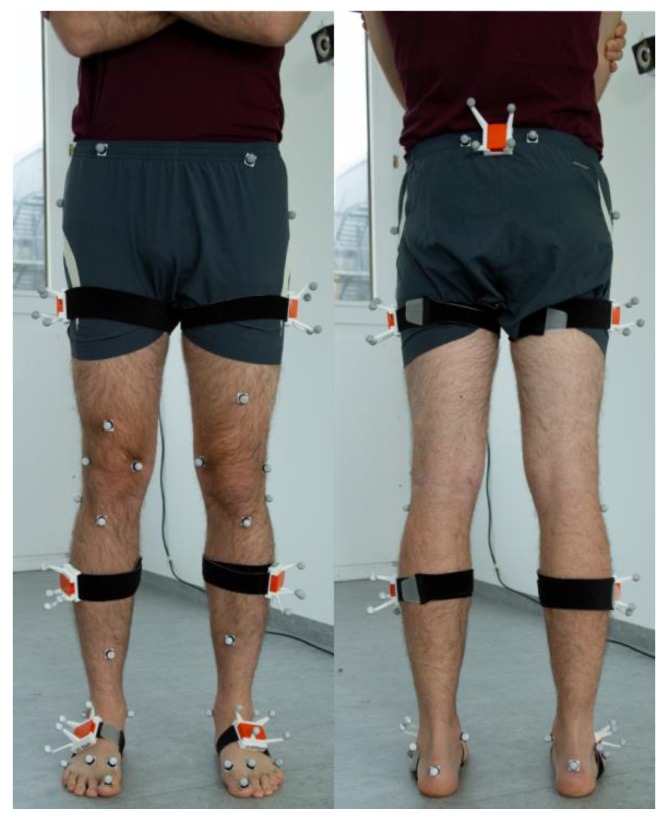
Schematic preparation of a subject with retroreflective markers and inertial measurement units (IMUs) inserted into rigid boxes equipped with additional markers. In the actual study, the markers on the pelvis were placed directly onto the skin. Furthermore, the subjects in the present study had to wear shoes.

The subjects were instrumented by means of seven IMUs (MTw Awinda, Xsens Technologies BV, Enschede, Netherlands) and 32 retroreflective markers, positioned on anatomical landmarks according to the marker protocol described by Leardini et al. [46]. To reduce the effect of soft tissue artefacts on the difference between the OMC data and the IMU data the IMUs were inserted into rigid boxes (RB) equipped with four additional markers. Figure 1 shows a schematic model of the marker and IMU positioning. In contrast to the depicted model, the subjects in the current study had to wear shoes for hygienic reasons. Therefore, the markers on the foot had to be positioned on the shoe, approximating the underlying anatomical landmarks.

First, the subjects performed the 2-step calibration described in [36]. The 2-step calibration consists of two adapted static postures according to [47] and is used for the IMU to segment (I2S) calibration. Second, the subjects had to walk along a walkway of seven meters for a maximum of six minutes. The 3D marker positions were recorded using 12 OptiTrack Prime 13 cameras and the software Motive 1.10 (OptiTrack, NaturalPoint, Inc., Oregon, USA). The IMU data was recorded using the Xsens software MVN Biomech 4.3.7. For the further calculations, only the raw accelerometer data and raw gyroscope data were considered. All records were taken simultaneously and were hardware-synchronized using a standard 5V transistor-transistor logic signal.

### 2.4. Data Processing

The resulting biases from Section 2.3 were subtracted prior to the processing of the raw IMU data. Based on the IMU raw data the 3D joint angle kinematics of the hip, knee and ankle as well as the 3D global pelvis rotation were calculated. However, only the hip and pelvis kinematics were considered for further procedure based on the selected features in Table 1. The same initialization and I2S calibration as reported previously in [36] were applied. Additionally, the segment lengths were scaled according to anthropometric tables and the body height. The segment coordinate systems and the joint centers were derived based on the segment lengths and a biomechanical human body model incorporating anatomical landmark positions described in [48]. For the segment orientation tracking an iterated extended Kalman filter (IEKF) approach according to [38] was used to fuse the gyroscope and accelerometer data. The same filter settings and tuning parameters as in [39] were incorporated.

The segment orientations based on the OMC data were estimated based on the RB orientations according to the recommendations of Visual 3D (C-Motion, Inc., Germantown, MD, USA). The IMU-based relative joint angle rotations as well as the OMC-based ones were calculated from the estimated segment orientations via Euler angle decomposition [49]. A detailed description of the IEKF can be found in [39]. Detailed descriptions of the IMU calibration, initialization as well as the biomechanical model building, can be found in [36]. The Euler angle decomposition was described in a supplementary file of [40].

Furthermore, the initial contact (IC) and terminal contact (TC) events were estimated according to [36] and based on that information the STP stride length, stride time, cadence and speed were calculated. The same approach for the calculation of the gait events and the STP was previously validated among the Control Group in [36], where only the validity of the STP was reported. Therefore, in the present study the joint angle kinematics of the Control Group, based on the raw IMU data from [36], were calculated in the same manner as for the THA Group.

### 2.5. Feature Validation—Statistical Analysis

In [39] the drift-free measurement of the IMU-based joint kinematics of the lower extremity, with the exception of the global pelvis rotation, was shown. However, in [39] it was also shown that the ROM of the global pelvis rotation was not affected by the drift. Therefore, in this study 18,000 frames of the record, of approximately 5 min, were considered for statistical analysis.

For further investigations the IMU-based joint angle waveforms were segmented into 100% gait cycle (GC) based on the IC information. Each GC of each subject was treated as an individual case. Outliers were detected based on the GC duration and removed if they were outside a boundary of the mean GC duration plus 2 times standard deviation.

The features described in Table 1 were then calculated for every GC of each subject in the THA Group as well as the Control Group. This approach resulted in a total of 1856 samples per feature (1402 labeled “healthy”, aka Control Group; 454 labeled “patient”, aka THA Group).

For comparison, the features were also calculated based on the OMC data, also segmented to 100% GC. In this case the IC was estimated based on the markers placed on the heel, toe and the pelvis and a custom written Matlab (Mathworks Inc., Natick, MA, USA) script incorporating an approach based on [50,51]. Here, feature Set 1 and 2 counted 1937 samples per feature (1404 labeled “healthy”, aka Control Group; 533 labeled “patient”, aka THA Group).

To validate the features, the following statistics were calculated based on every GC of each subject: the root mean squared error (RMSE) plus 95% confidence interval (CI), the mean absolute error (MAE) plus 95% CI and r as well as the coefficient of determination (r^2^).

Additionally, the range of motion error (ROME) plus 95% CI and the coefficient of multiple correlations (CMC) were calculated for the 3D joint kinematics of the pelvis and hip. In the case of waveforms rather than single values the CMC was preferred to r according to [52].

A two-sample independent t-test was calculated to find significant differences between the THA Group and the Control Group in the RMSE of the features of Sets 1/2 and the ROME of the pelvis and hip joint kinematics. The chi square goodness of fit test was used to check for a normal distribution in the data. All statistics were conducted in Matlab 2019a and 2019b.

### 2.6. Classification Algorithm

Based on the information reported in Section 1, a SVM was deemed the appropriate tool for the present study. Figueiredo et al. [29] stated that SVMs are well capable of dealing with the non-linear character of human gait and represent an accurate classifier in recognition tasks concerning impaired and non-impaired gait.

First, the SVM algorithm was trained on the IMU-based features. Second, the SVM algorithm was also trained on the same features based on OMC data

**Figure 2:**
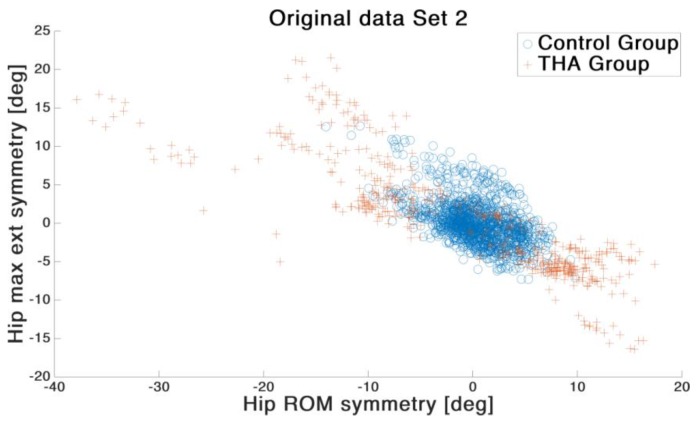
Examplary data from Set 2 showing on the X-axis the hip ROM symmetry and on the Y-axis the hip maximum extension symmetry. Blue circles indicate the Control Group; red crosses indicate the total hip arthroplasty (THA) Group. The plot indicates a non-linear differentiation of the two groups.

Figure 2 shows the comparison of two representative features. Qualitative examination revealed that the two groups showed a non-linear relation within all features. Therefore, in this study an SVM with Gaussian RBF kernel was applied. For the model training, the Classification Learner^®^ app from Matlab 2019b was used. In every case the Box Constrained level was set to 1.7 and the Kernel scale was set to auto. Standardize data was set to true. A 12-fold cross-validation was employed to prevent overfitting.

### 2.7. Classification—Statistical Analysis

To compare and interpret the results of the different classifier models the following measures were calculated as reported in [53]:

The accuracy (ACC) was calculated based on the true positive rate (TP), true negative rate (TN), false positive rate (FP) and false negative rate (FN).

(1) $$ACC\;\left(\%\right)=\frac{TN+TP}{TP+TN+FP+FN}*100\%$$

The sensitivity (SEN) was calculated based on the TP and FN.

(2) $$SEN\;\left(\%\right)=\frac{TP}{TP+FN}*100\%$$

The specificity (SPEC) was calculated based on the TN and FP.

(3) $$SPEC\;\left(\%\right)=\frac{TN}{TN+FP}*100\%$$

Additionally, the AUC [30] was calculated.

**Figure 3:**
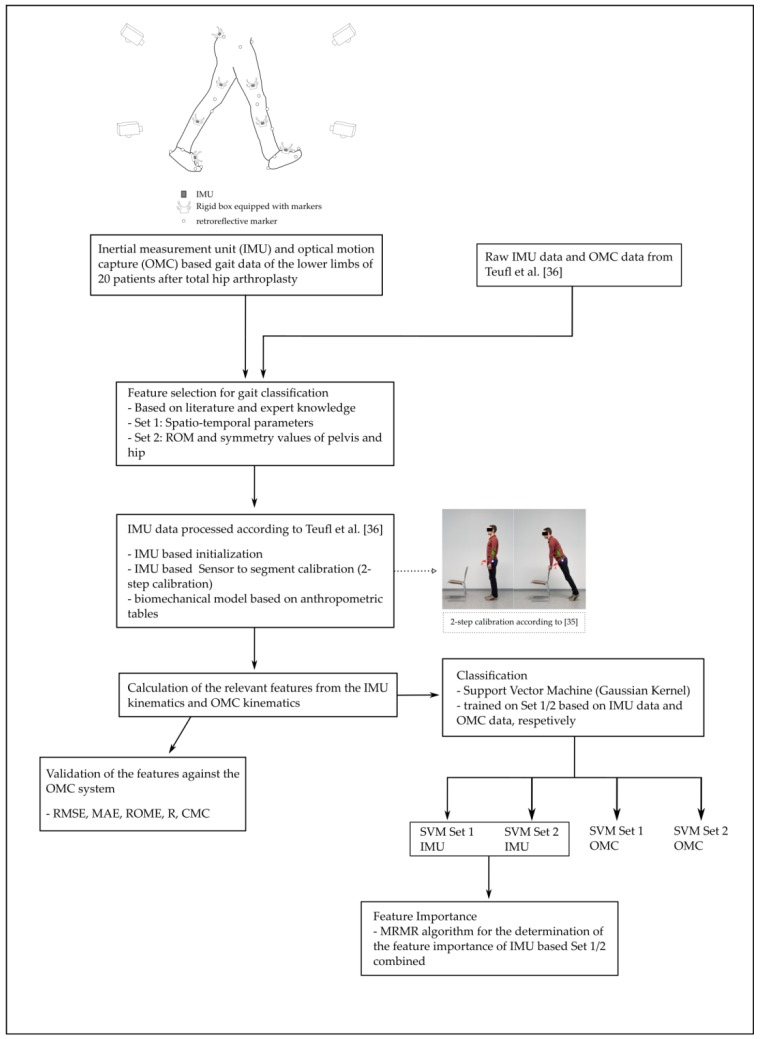
Workflow of the present study.

Figure 3 summarizes the framework of the present study.

## 3. Results

### 3.1. Feature Validation

In this section the validity of the features of Set 1 and Set 2 as well as of the ROM of the pelvis and hip in three dimensions are presented for the THA Group and the Control Group, respectively. In the following, the values of the statistical measures are considered the mean over all subjects.

The validation process revealed a high accuracy of the features measured with the IMU system. The RMSE of the feature Set 2 was below 1.3° in both groups. Significant differences between the groups were found in the hip ROM symmetry and the pelvis transversal ROM. Details are shown in Table 2 and Table 3.

The ROME of the global pelvis and the hip joint in the sagittal, frontal and transverse plane revealed values from 0.36° to 2.70° in both groups. Significant differences between the groups in the ROME were evident in the left and right hip flexion and in the pelvis obliquity as well as pelvis rotation. Details are shown in Table 4.

The validation of the STP (feature Set 1) of the Control Group can be found to its full extend in [36]. Therefore, in Table 5 the results of the validation of feature Set 1 are shown only for the THA group. Significant differences between the RMSE in the STP of the THA Group and the corresponding results of the Control Group were found in all four parameters.

### 3.2. Calssification

**Figure 4:**
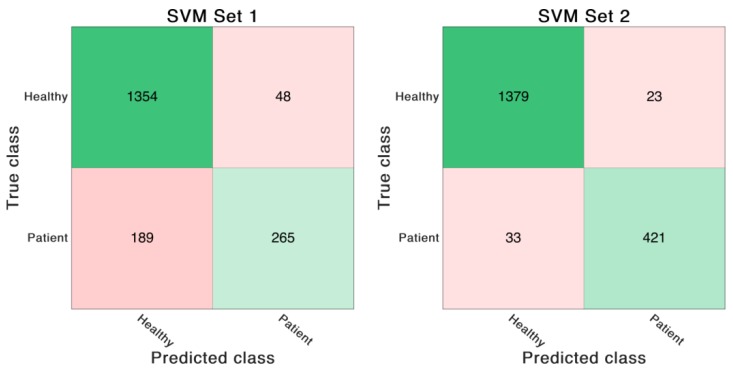
Confusion matrices of the SVM trained on IMU-based feature Set 1 and 2. The numbers in the matrices indicate correctly or incorrectly classified GC.

**Figure 5:**
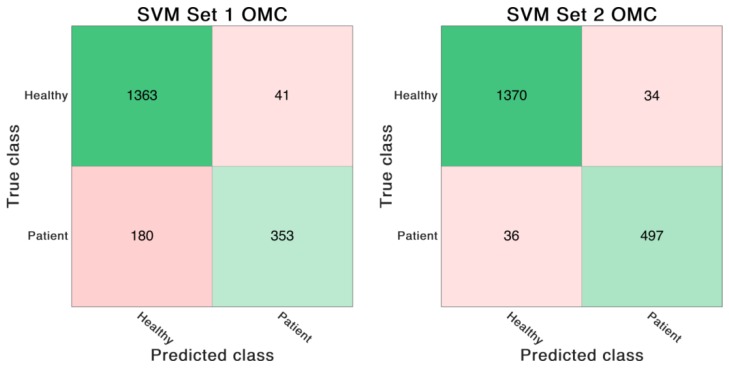
Confusion matrices of the SVM trained on OMC-based feature Set 1 and 2. The numbers in the matrices indicate correctly or incorrectly classified GC.

**Figure 6:**
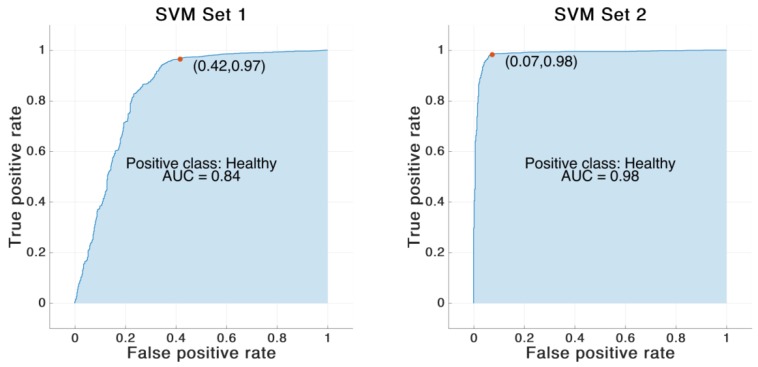
Receiving operating characteristic (ROC) curve for the SVM model trained on IMU-based feature Set 1 and 2. The red dot marks the performance of the corresponding model. The blue area indicates the area under the curve (AUC).

**Figure 7:**
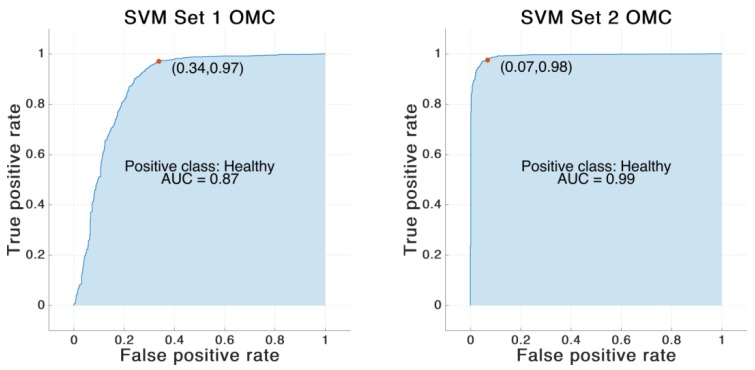
ROC curve for the SVM model trained on OMC-based feature Set 1 and 2. The red dot marks the performance of the corresponding model. The blue area indicates the AUC.

The SVM classifier trained on the feature Set 1 reached an accuracy of 87.2%. In contrast, the SVM classifier trained on feature Set 2 achieved an overall accuracy of 97.0%. The same SVM model was also trained on the same features derived from optical data. In that case the classifier trained on feature Set 1 showed an accuracy of 88.6%. The classifier trained on feature Set 2 revealed an accuracy of 96.4%. See Table 6 and Figure 4, Figure 5, Figure 6 and Figure 7 for detailed information on the results of the four classifier variations.

### 3.3. Feature Importance

**Figure 8:**
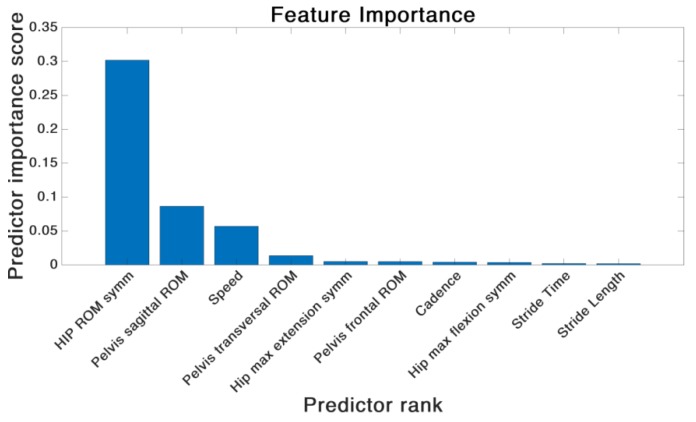
Ranking of the combined features from Set 1 and 2 based on IMU data after employing a minimum redundancy maximum relevance (MRMR) feature ranking algorithm.

In the case of the IMU driven SVM classifier a MRMR algorithm was employed to calculate the feature importance of the combined features of Set 1 and Set 2. Figure 8 shows the outcome of the calculation.

The ranking of the feature weights revealed that the hip ROM symmetry contributed most to the separation of the two groups. Overall, three out of the top-four ranked features belonged to the feature Set 2.

A post-examination revealed an accuracy of 95.7% of the SVM classifier trained on the four most important features.

### 3.4. Feature Correlation

The calculation of the correlation between the features in Set 1 and Set 2, respectively, revealed that the feature speed was correlated with stride time (−0.78) and stride length (0.80). Furthermore, in Set 2 the feature hip ROM symmetry showed correlations with hip maximum flexion symmetry (0.71) and hip maximum extension symmetry (−0.76). See Table 7 and Table 8 for details.

## 4. Discussion

The present examination evaluated the applicability of features taken from the IMU-based 3D gait kinematics of the lower body for the discrimination of the gait of patients after THA and a healthy control. Therefore, special features were chosen according to the literature and expert knowledge. A first step was to validate the accuracy of the measured features in comparison to an OMC system. As a second step, the features were divided into two groups of features, STP and joint kinematics, due to the different kind of measurement approach to these variables. Then, an SVM model was trained on both of the feature sets, first on the IMU-derived features and second, for the sake of comparison, on the OMC derived features.

### 4.1. Feature Selection

Laroche et al. [1] reported that in gait analysis there is too many data recorded and it is therefore important to identify relevant features. In the present study no common feature selection algorithms were employed. As mentioned in Section 1 it was the aim to select meaningful, intelligible features from among the IMU kinematic data based on the literature and expert knowledge. It was important that the selected features are mostly independent of uncertainties in the measurement method. To date, in IMU systems one major concern is the I2S calibration. In [37] it was shown that errors in the I2S orientation were linearly transferred into the segment orientation estimation and therefore directly affect the calculated joint angles. Common IMU systems as well as the present system rely in their I2S calibration procedure on pre-defined poses, e.g., n-pose or T-pose [54]. However, in [55] it was shown that the n-pose can differ up to 15° from the assumed zero position in the joints of the lower limb. Additionally, the work revealed that in older, impaired subjects the deviations tend to increase, mainly in the frontal plane.

Considering these uncertainties in the I2S calibration process, high absolute deviations between the OMC-based joint angle waveforms and the IMU-based joint angle waveforms, i.e., static offsets, can be expected. Actually, in the present evaluation offsets in the 3D joint kinematics of the hip and pelvis up to 12.52° were found. A detailed examination and fragmentation of these errors is determined for future work.

However, differences between absolute values, expressed here as symmetry between left and right lower limb and the ROM, are considered unaffected by offsets, as long as they are static. To further prove this, the individual features as well as the ROM of the joints of interest were in this study validated against the OMC system.

### 4.2. Feature Validation

To the knowledge of the authors this is the first study to validate parameters from among IMU-based joint kinematics of the human gait, i.e., the features in Set 2, designed for training a classification model. It is deemed essential to prove the accuracy of features prior to its employment in a ML algorithm.

In the validation of the feature Set 2, low errors (<1.3°) were found in both groups. However, there were significant differences between the two groups in the RMSE of the hip ROM symmetry and the pelvis transversal ROM. Also in the ROME of the 3D joint kinematics of the hip and pelvis appeared significant differences between the groups (left and right hip flexion, pelvis obliquity and rotation). Concerning the significant differences between the groups in the RMSE and ROME of some features and joint angles one has to consider a few differences in the measurement set-up of both groups. First, there were non-system related differences like the measurement location, the footwear and finally the subject’s physique. The THA Group had to wear shoes due to hygienic reasons and walked on an artificial walkway incorporating force plates. The walkway exhibited a slight inclination, which was evident in the raw marker data of the OMC system. The subjects of the control group walked bare footed and on a normal floor. Further, the two groups differed, as it was intended, in their body stature. The THA group showed an average body mass index (BMI) of 27.16, whereas the control group had an average BMI of 22.49.

Zügner et al. [27], who validated the accuracy of the IMU-based pelvis, hip, knee and ankle joint angle of the sagittal plane within a group of 49 patients after THA, found a significant error in the hip sagittal ROM of about 3°. In the pelvic sagittal ROM an error of approximately 0.5° was found in their study, similar to the present findings.

Zhang et al. [17] evaluated the accuracy of an IMU system in 10 healthy subjects. They found a ROME in the 3D kinematics of the hip of 2.47° to 4.83°.

It was shown that the ROM can be measured with high accuracy in the presence of variations in the calibration or the physique of the subjects. It is considered important that the ROME showed low values in both groups, since the ROM is regarded an essential outcome in the evaluation of the rehabilitation progress [56].

The estimation of the STP within the THA group showed valid results comparable to those results found previously within the control group [36] and to results from studies employing alternative systems for the STP estimation [26,57].

Kluge et al. [26] examined the validity of STP in subjects with Parkinson disease. Their reported mean errors of the temporal parameters were slightly higher compared to the present study. The results of the stride length revealed a smaller error (−0.001 m). They also evaluated the validity among a healthy population. Interestingly they found a higher error in the stride length concerning that group (−0.016 m). That did not apply for the current system.

Bertoli at al. [57] also examined the validity of the STP of patients with Parkinson disease, mildly cognitive impaired subjects as well as older, healthy subjects from different clinical facilities. For the stride length, they reported a mean error of −0.001 m to −0.014 m in the different clinics. Therefore, the present findings (mean error of 0.009 m) lie well within that range. The temporal parameters described by Bertoli et al. [57] show a mean error of under 0.001 s for stride time. Therefore, their results show a similar outcome compared to the current findings (mean error of 0.002 s). Note, the referenced works used a sampling frequency above 100 Hz whereas the IMUs in the present study were recording at a frequency of 60 Hz.

In feature Set 1, significant differences between the RMSEs of the THA Group and the Control Group were evident in all four parameters. That might be due to the same reasons as mentioned above concerning feature Set 2. However, the fact that the algorithm for the event detection was developed on young and healthy subjects and has not been modified for the present study might also have influenced the outcome of the STPs in the THA Group.

In summary, it can be stated that static offsets between the OMC and the IMU system were evident. These offsets might be explained by deviations from the neutral-zero calibration position or uncertainties in the segment length estimation and based on that erroneous joint center estimates. It was shown that a misalignment between joint centers and joint axes could lead to kinematic cross talk [28]. However, the selected features of Sets 1 and 2 as well as the ROM seemed unaffected by these errors. Nevertheless, these possible sources of errors should be erased in the future by employing an automated self-calibration, independent from poses, pre-defined movements as well as a strict sensor to segment assignment. A proof of concept was delivered recently [58,59].

Furthermore, a more individualized biomechanical model could improve the estimation of segment lengths and consequently the joint centers. However, an alternative approach, in regard to the scaling approach used in the current study and in [36], should support the idea of a mobile and flexibly applicable system. Therefore, the creation of a biomechanical model of the lower body based on a single-view depth camera image was recently proposed [60].

### 4.3. Classification

It was the aim of this part of the study to evaluate the usability of the above validated features in the classification of impaired and non-impaired gait. For this purpose the two subsets of relevant kinematic parameters were used to train an SVM model for separating the gait of patients after THA and of healthy subjects, respectively.

The classifier trained on feature Set 1, consisting of commonly used STP, showed a high accuracy (87.2%). However, the classifier trained on feature Set 2 showed an even higher accuracy (97.0%), using features based on 3D joint kinematics. Further, in the present study the same features were calculated based on the OMC data and employed to train a similar SVM model. This was done to prove the independence of the SVM model regarding the measurement approach that was used to derive the features. As expected the classifiers trained on feature Set 1/2 based on OMC data revealed a similar accuracy (Set1: 88.6°, Set 2: 96.4°) compared to the IMU-based classifier.

In summary, the model trained on feature Set 2 revealed better results. However, also the accuracy of the model trained on the feature Set 1 showed a satisfying accuracy. The post-hoc analysis of the feature importance showed the impact of the features of Set 2 on the classifier accuracy. The hip sagittal ROM proved to be the most important factor. This result supports the findings of Pietschmann et al. [41] who addressed the hip sagittal plane movement as a key parameter in their attempt to improve the gait pattern of patients after THA via acoustic feedback.

The features of Set 1 were shown to have less impact on the accuracy of the classifier despite the fact that the RMSE in all of the STP of feature Set 1 were significantly different between the two groups. Therefore, a reason could have been a high correlation between the features in Set 1. However, it was shown that only the feature speed was correlated with stride time and stride length. That was not unexpected since speed was calculated using Stride Time and Stride Length. The same goes for the features hip ROM symmetry, hip maximum flexion symmetry and hip maximum extension symmetry of Set 2. Since the Hip ROM symmetry contains information of the hip flexion and extension, these features were also correlated.

Therefore, the hip ROM symmetry and speed can be considered to contain more information compared to the remaining features. That was also proven in the feature importance calculation where the aforementioned parameters were within the top three ranked features.

There is another advantage in measuring the joint angle kinematics rather than the STP alone. Asymmetries in the STP, e.g., stride length, could be reduced by introducing compensatory mechanisms like an increased pelvic extension in late stance of the operated limb to compensate for a lack of hip extension [61]. Therefore, in an analysis a symmetry between operated and non-operated limb would be evident in the parameter stride length, whereas asymmetries could still be found in the pelvis and hip joint angle [62]. In that case, disadvantageous gait deviations can only be detected by evaluating the complete 3D gait kinematics of the lower body.

However, the examination revealed that with the kinematic information of the pelvis and the hip as well as the gait speed a classification of the gait can be achieved with an accuracy of 95.7%. In this study a full lower body set up of IMUs, i.e., seven IMUs, was initially used. The results imply that a reduced sensor set up might result in the same outcome. Employing three IMUs, mounted on the pelvis and thighs, would be sufficient to deduce the gait speed as well as the 3D global pelvis motion and the 3D joint kinematics of the hip. However, that demands an estimation of the gait events using the IMU mounted on the pelvis. In general it was shown that the gait events and the step length can be deduced from a pelvis-mounted IMU [63]. Furthermore, it has not been evaluated yet if the joint kinematics of the hip as well as the estimation of the 3D pelvis motion might suffer from a sensor reduction, using the present system. However, that is intended for future work.

It has to be stated that the results of the SVM classifier reported in this article are only valid for the specific data set used in this study. Consider that the present gait data was recorded under laboratory conditions and from a rather small sample size. However, through the extended recording time a considerable number of GCs could be regarded for the evaluation. Future work has to investigate the usability of the described IMU system as well as the utility of the proposed SVM classifier based on real-world and non-standardized recorded data.

A further limitation is the fact that the two groups were not age-matched. Strictly speaking the difference between the groups could have been due to age alone. However, Boyer et al. [64] reported that the changes in the hip kinematics due to age are rather small. Moreover, they stated that the hip ROM is slightly higher in the elderly group. That would indicate an even higher difference between subjects after THA and an age-matched control. Besides, there is no evidence that the asymmetry between the lower limbs increases at an advanced age. Nevertheless, a comparison of the THA Group with an age-matched control group would have strengthened the present outcome.

Furthermore, Ewen et al. [65] stated in their review on post-operative gait analysis after THA that the rehabilitation progress reaches a peak six months after the surgery. The patients in the present study were tested only a short time after THA, meaning they were in the midst of their stationary rehabilitation phase. Consequently a significant progress would have been expected within the next weeks. A possible feedback system based on IMU data would take up approximately after a stationary rehabilitation. Therefore, it has to be examined if the asymmetries and gait deviations at that date are distinctive enough for successful classification using the model and features employed in the present study. On the other hand, several studies showed that significant differences between operated and non-operated side as well as a healthy control group exceed six months and more [7,11].

## 5. Conclusions

In summary, the present work describes an IMU system that accurately measures the ROM and special features of interest of the lower limb 3D kinematics of the human gait, in patients after THA as well as in a healthy population. Although static offsets are to be expected, it was shown that the symmetry values based on joint kinematics, the STP and, especially, the important ROM could be measured with satisfying accuracy among subjects of differing physique. However, future work will aim at the improvement of the calibration process as well as a refinement of the biomechanical model.

Furthermore, the current study proved that the described system can be successfully used to classify the gait of impaired and non-impaired subjects, employing a SVM model and elaborated features of the joint kinematics. However, this approach has to be tested for its functionality in non-standardized settings.

As mentioned in [36], it is the superior goal of the authors of the present work to design a mobile IMU system that delivers 3D gait kinematics, joint angles as well as a wide range of STP, based on which feedback on the individual gait pattern can be provided to the user independent of the location and beyond the standard rehabilitation period. In the opinion of the authors, a further step towards that goal was achieved in this work by defining valid and intelligible features from IMU-based gait kinematics that are sensitive to the subject’s impairment and, therefore, seem promising as control parameters in a future feedback system.

## Figures and Tables

**Table 1:** Description of the two feature sets used for training the support vector machine (SVM) model.

**Set 1**	**Definition**
Stride Length [m]	Distance between the calcaneus positions of one foot projected on the ground at two consecutive ipsilateral initial contacts (IC)
Stride time [s]	Period between two consecutive ICs of the ipsilateral foot
Cadence [steps/min]	60 divided by the time from the IC of the ipsilateral foot to the IC of the contralateral foot
Speed [m/s]	Stride length divided by Stride time
**Set 2**	
Hip range of motion (ROM) symmetry [deg]	Difference between left and right sagittal hip ROM per gait cycle (GC)
Hip maximum flexion symmetry [deg]	Difference between left and right hip maximum flexion per GC
Hip maximum extension symmetry [deg]	Difference between left and right hip maximum extension per GC
Pelvis sagittal ROM [deg]	ROM of the pelvis in the sagittal plane per GC
Pelvis frontal ROM [deg]	ROM of the pelvis in the frontal plane per GC
Pelvis transversal ROM [deg]	ROM of the pelvis in the transversal plane per GC

**Table 2:** Validation results of the feature Set 2 within the THA Group. Shown are the root mean squared error (RMSE) ± standard deviation (SD) (95% confidence interval (CI)), the mean absolute error (MAE) ± SD (95% CI), the pearson correlation coefficient (r) ± SD and the coefficient of determination (r^2^). An asterisk indicates a significant difference between the THA Group and the Control Group. The corresponding *p*-values are given.

	RMSE ± SD (95% CI) [deg]	*p*-Value	MAE ± SD (95% CI) [deg]	r ± SD	r^2^
Hip ROM symmetry	1.16 ± 0.92 (0.32–1.23) *	0.008	0.48 ± 0.69 (−0.15–0.54)	0.90 ± 0.21	0.81
Hip maximum flexion symmetry	1.21 ± 0.81 (0.39–1.20)	0.214	0.51 ± 0.59 (−0.06–0.52)	0.48 ± 0.56	0.23
Hip maximum extension symmetry	1.24 ± 1.18 (0.11–1.29)	0.092	0.52 ± 0.71 (−0.15–0.56)	0.71 ± 0.50	0.50
Pelvis sagittal ROM	0.40 ± 0.25 (0.14–0.39)	0.054	0.15 ± 0.16 (−0.02–0.14)	0.94 ± 0.18	0.88
Pelvis frontal ROM	0.39 ± 0.32 (0.14–0.45)	0.083	0.16 ± 0.24 (−0.06–0.18)	0.95 ± 0.16	0.90
Pelvis transversal ROM	1.25 ± 0.80 (0.59–1.38) *	0.000	0.47 ± 0.50 (−0.05–0.45)	0.91 ± 0.24	0.83

* Significant difference at *p*-value < 0.05.

**Table 3:** Validation results of the feature Set 2 within the Control Group. Shown are the RMSE ± SD (95% CI), MAE ± SD (95% CI), r ± SD and r^2^.

	RMSE ± SD (95 % CI) [deg]	MAE ± SD (95% CI) [deg]	r ± SD	r^2^
Hip ROM symmetry	0.52 ± 0.39 (0.17–0.50)	0.21 ± 0.28 (−0.04–0.20)	0.88 ± 0.07	0.77
Hip maximum flexion symmetry	0.83 ± 1.11 (−0.09–0.87)	0.45 ± 0.95 (−0.31–0.51)	0.83 ± 0.18	0.67
Hip maximum extension symmetry	0.68 ± 0.85 (−0.02–0.71)	0.34 ± 0.70 (−0.21–0.40)	0.74 ± 0.20	0.55
Pelvis sagittal ROM	0.24 ± 0.21 (0.07–0.26)	0.09 ± 0.12 (−0.02–0.08)	0.98 ± 0.03	0.96
Pelvis frontal ROM	0.25 ± 0.13 (0.14–0.25)	0.09 ± 0.10 (0.01–0.09)	0.99 ± 0.06	0.98
Pelvis transversal ROM	0.36 ± 0.25 (0.19–0.41)	0.12 ± 0.15 (−0.00–0.13)	0.99 ± 0.03	0.98

**Table 4:** Validation results of the 3D range of motion (ROM) of the left (LT) and right (RT) hip and pelvis in both groups. Shown are the ROM error (ROME) ± SD (95% CI) and the coefficient of multiple correlation (CMC) ± SD. An asterisk indicates a significant difference between the THA Group and the Control Group. The corresponding *p*-values are given.

	THA Group			Control Group	
	ROME [deg] ± SD (95% CI)	*p*-Value	CMC ± SD	ROME [deg] ± SD (95% CI)	CMC ± SD
LT Hip–Abduction	0.89 ± 0.60 (0.46–1.13)	0.742	0.76 ± 0.24	0.83 ± 0.48 (0.57–0.98)	0.87 ± 0.16
LT Hip–Rotation	0.84 ± 0.36 (0.63–1.03)	0.226	0.69 ± 0.24	1.05 ± 0.62 (0.63–1.15)	0.66 ± 0.24
LT Hip–Flexion	0.85 ± 0.46 (0.49–1.00) *	0.000	0.73 ± 0.20	2.70 ± 0.97 (2.32–3.14)	0.93 ± 0.12
RT Hip–Abduction	1.10 ± 0.55 (0.85–1.38)	0.053	0.83 ± 0.19	0.80 ± 0.44 (0.45–0.83)	0.93 ± 0.06
RT Hip–Rotation	0.98 ± 0.60 (0.45–1.03)	0.241	0.60 ± 0.30	1.20 ± 0.60 (0.79–1.30)	0.71 ± 0.23
RT Hip–Flexion	1.20 ± 0.60 (0.92–1.50) *	0.001	0.82 ± 0.21	2.11 ± 1.01 (1.44–2.30)	0.93 ± 0.11
Pelvis–Obliquity	0.36 ± 0.24 (0.18–0.41) *	0.000	0.88 ± 0.11	0.73 ± 0.35 (0.49–0.79)	0.90 ± 0.11
Pelvis–Flexion	0.51 ± 0.17 (0.43–0.60)	0.728	0.44 ± 0.18	0.56 ± 0.59 (0.15–0.65)	0.52 ± 0.25
Pelvis–Rotation	0.98 ± 0.46 (0.66–1.10) *	0.044	0.58 ± 0.30	0.75 ± 0.27 (0.66–0.88)	0.65 ± 0.22

* Significant difference at *p*-value < 0.05.

**Table 5:** Summary of the results of the validation of the feature Set 1 for the THA group. Shown are the RMSE ± SD (95% CI), the MAE ± SD (95% CI), r and r^2^. An asterisk indicates a significant difference between the THA Group and the Control Group. The corresponding *p*-values are given.

	RMSE ± SD (95% CI)	*p*-Value	MAE ± SD (95% CI)	r	r^2^
Stride Length [m]	0.05 ± 0.03 (0.03–0.05) *	0.007	0.06 ± 0.04 (0.03–0.06)	0.78	0.61
Stride Time [s]	0.04 ± 0.02 (0.02–0.04) *	0.000	0.05 ± 0.02 (0.02–0.05)	0.91	0.83
Cadence [steps/min]	3.85 ± 2.50 (1.77–4.43) *	0.000	4.86 ± 2.90 (2.27–5.36)	0.54	0.29
Speed [m/s]	0.04 ± 0.02 (0.02–0.04) *	0.000	0.05 ± 0.03 (0.03–0.06)	0.84	0.71

* Significant difference at *p*-value < 0.05.

**Table 6:** Results of the different SVM models trained on feature Set 1 and Set 2. “OMC” indicates the support vector machine (SVM) model trained on the features calculated based on the optical motion capture system.

	SVM Set 1	SVM Set 2	SVM Set 1 OMC	SVM Set 2 OMC
Accuracy [%]	87.2	97.0	88.6	96.4
Sensitivity [%]	87.8	97.7	88.3	97.4
Specificity [%]	84.7	94.8	89.6	93.6
Area under the curve	0.84	0.98	0.87	0.99

**Table 7:** Correlation matrix for the features of Set 1. Shown are the values for r.

	Stride Time	Stride Length	Cadence	Speed
Stride Time	1.00	x	x	x
Stride Length	−0.33	1.00	x	x
Cadence	−0.53	0.10	1.00	x
Speed	−0.78	0.80	0.46	1.00

**Table 8:** Correlation matrix for the features of Set 2. Shown are the values for r.

	Hip ROM Symm.	Hip Max Extension Symm.	Hip Max Flexion Symm.	Pelvis Sagittal ROM	Pelvis Frontal ROM	Pelvis Transversal ROM
Hip ROM symm.	1.00	x	x	x	x	x
Hip max extension symm.	−0.76	1.00	x	x	x	x
Hip max flexion symm.	0.71	−0.09	1.00	x	x	x
Pelvis sagittal ROM	−0.30	0.28	−0.16	1.00	x	x
Pelvis frontal ROM	0.16	-0.08	0.16	0.02	1.00	x
Pelvis transversal ROM	−0.17	0.12	−0.14	0.26	0.29	1.00
